# Supplementary material for: Migraine Genetic Susceptibility Does Not Strongly Influence Migraine Characteristics and Outcomes in a Treated, Real-World, Community Cohort
Source: J Clin Med. 2025 Jan 16;14(2):536. doi: 10.3390/jcm14020536 (PMC11765864; doi:10.3390/jcm14020536)
Supplement: Supplementary file 1 [file jcm-14-00536-s001.zip › jcm-3316257-supplementary.pdf]

Supplemental Item **Page**

Table S1 DodoNA migraine-toolkit data elements 2
Table S2 Association of migraine PGS004799 with migraine characteristics after correction for multiple tests 5
Table S3 Measures/symptoms differing across deciles of PGS004799 in unadjusted analyses 7

Table S1: DodoNA migraine-toolkit data elements[a]

Data Element	Recorded As
**Medical History**	
Encounter date	Date
Age	Years
Body mass index (BMI)	BMI
Sex	M/F
Education	Years
Family history: epilepsy, hypertension, migraine, neurological disease, Parkinson's disease, stroke	Presence / absence of each in enumerated first-degree relatives
**History of Migraine Attacks**	
Age at onset (AAO)	Years
Frequency	<1/mo, 1-3/mo, 1/wk, 2-3/wk, >3/wk, daily, constant
Estimated lifetime total	<5, 5-10, 11-50, 51-100, >100
Location	Unilateral/bilateral
Details	Primary headache; Left, right, alternating (unilateral); frontal, orbital, parietal, temporal, occipital, holocephalic (bilateral)
Quality	None, throbbing, pressure, vise-like, stabbing, dull aching, imploding, exploding
Severity	0-10
Associated symptoms	None, nausea, vomiting, sensitivity to light, noise, smell; change in vision, dizziness, fatigue, face-flushing, red-eye, ptosis, runny nose/eyes, allodynia, ringing in ears, neck stiffness
Average duration	<5 min, 5-15 min, 15-60 min, 1-4 hr, 4-24 hr, 1-3 days, >3 days, constant
Timing	Continuous, any time, waking, morning, afternoon, evening, night, during sleep, weekends, peri(menstrual)
Triggers	None, weather, altered sleep, stress, skipped meals, smells, sounds, menstruation, alcoholic beverages, foods, caffeine, chocolate, Nutrasweet, MSG, cured meat, onion, aged cheese, banana, nuts, other
Time to peak intensity	<5 min, 5-15 min, 15-60 min, 1-4 hr, >4 hr
Aura symptoms	None, visual, dysarthria, aphasia, sensory, motor
Aura duration	<5 min, 5-60 min, 1-3 hr, 3-24 hr, >24 hr
Influences	None, abortive medication, cold compresses, heat, massage, relaxation, sleep, other
Exacerbating measures	None, routine physical activity, exercise, coughing, sneezing, straining, bending, change in position, other
Temporal course	Progressing, improving, static, resolved
Emergency department (ED) visits for headache	Ever, number in past year (0 – 12+)
Hospitalizations for headache	Ever, number in past year (0 – 12+)
**Clinical Impression** (diagnosis using International Classification of Headache Disorders 3rd Ed. criteria)	
Headache disorder	Present / absent
Primary headache disorder	Present / absent
Migraine	Present / absent
Tension-type headache	Present / absent

Cluster headache / other trigeminal autonomic cephalalgias	Present / absent
Other primary headache	Present / absent
Migraine Subtype	
Migraine with aura (MA)	Present / absent
Migraine without aura (MoA)	Present / absent
Childhood periodic syndromes that are common precursors of migraine	Present / absent
Retinal migraine	Present / absent
Probable migraine	Present / absent
If migraine with aura -	
Typical aura with migraine headache	Present / absent
Typical aura with non-migraine headache	Present / absent
Typical aura without headache	Present / absent
Familial hemiplegic migraine	Present / absent
Sporadic hemiplegic migraine	Present / absent
Basilar-type migraine	Present / absent
Migraine with brainstem aura	Present / absent
Chronic migraine	Present / absent, first occurrence (year)
Complications of migraine	
Migrainous infarction	Present / absent, first occurrence (year)
Status migrainosus	Present / absent, first occurrence (year)
Persistent aura without infarction	Present / absent, first occurrence (year)
Migraine-triggered seizures	Present / absent, first occurrence (year)
Secondary headache disorder	Presence / absence
Head and/or neck trauma	Presence / absence
Cranial and/or cervical vascular disorder	Presence / absence
Non-vascular intracranial disorder	Presence / absence
High CSF pressure	Presence / absence
Low CSF pressure	Presence / absence
Substance or its withdrawal	Presence / absence
Headache induced by acute substance use / exposure	Presence / absence
Medication overuse headache	Presence / absence
Infection	Presence / absence
Disorder of homeostasis	Presence / absence
Disorder of cranium, neck, other facial/cranial structures	Presence / absence
Psychiatric disorder	Presence / absence
Cranial neuralgias	Presence / absence
Trigeminal neuralgia	Presence / absence
Tolosa Hunt syndrome	Presence / absence
Supra orbital neuralgia	Presence / absence
Occipital neuralgia	Presence / absence
Herpes zoster	Presence / absence
Recurrent painful ophthalmoplegic neuropathy	Presence / absence
Other	Presence / absence
Co-morbidities: anxiety, asthma, bipolar disorder, depression, epilepsy, fibromyalgia, hypertension, insomnia, obesity, post-traumatic stress disorder, restless leg syndrome, sleep apnea, stroke, vertigo	Presence / absence of each
Abortives	Type / name

Number pills taken per month	Presence / absence
2-hr response	Yes / no / unknown
2-hr pain free	Yes / no / unknown
24-hr pain free	Yes / no / unknown
Loss of responsiveness	Yes / no / unknown
Rescue med	Yes / no / unknown
Preventives	Type / name
Active	Yes / no / unknown
Improved headaches	Yes / no / unknown
Tolerated	Yes / no / unknown
Complementary and alternative therapies	Type / name
Active	Yes / no / unknown
Improved headaches	Yes / no / unknown
Tolerated	Yes / no / unknown
Interventional therapies	Type / name
Active	Yes / no / unknown
Improved headaches	Yes / no / unknown
Tolerated	Yes / no / unknown
**Objective Test Scores**	
Migraine Disability Assessment (MIDAS)	Scores on questions 1-5, total score, A, B
Migraine-Specific Quality of Life (MSQ)	Role-function restrictive (RR), role-function-preventive (RP), emotional function (EF) scores, total score
Center for Epidemiologic Studies Depression Scale (CES-D)	Score
Insomnia Severity Index (ISI)	Score
Generalized Anxiety Disorder 7-item (GAD7)	Score

[a]Sex, education, family history and AAO were collected only at study enrollment. All other data elements were collected at study enrollment and at each annual follow-up.

Table S2: Association of migraine PGS004799 with migraine characteristics after correction for multiple tests[a]

Rank	Measure/Characteristic/Symptom	Original *p*	Critical value	Benjamini-Hochberg Adjusted *p* at Q = 0.05
1	photophobia	**0.001**	0.001	**0.021**
2	stabbing	**0.001**	0.002	**0.021**
3	phonophobia	**0.016**	0.004	0.224
4	emesis	**0.033**	0.005	0.234
5	ISI score at baseline	**0.038**	0.006	0.234
6	unilateral headache	**0.039**	0.007	0.234
7	nausea	**0.039**	0.008	0.234
8	severity	0.052	0.010	0.273
9	ln(MIDAS-A) at follow-up	0.123	0.011	0.574
10	ln(MIDAS-A) at baseline	0.179	0.012	0.583
11	time to peak intensity	0.18	0.013	0.583
12	osmophobia	0.199	0.014	0.583
13	neck stiffness	0.211	0.015	0.583
14	frequency at baseline	0.22	0.017	0.583
15	bilateral headache	0.221	0.018	0.583
16	any aura	0.227	0.019	0.583
17	triptan responsive	0.236	0.020	0.583
18	visual aura	0.281	0.021	0.656
19	migraine without aura	0.344	0.023	0.724
20	fatigue	0.345	0.024	0.724
21	migraine with and without aura	0.385	0.025	0.745
22	ISI score at follow-up	0.39	0.026	0.745
23	MSQ score at follow-up	0.456	0.027	0.811
24	allodynia	0.478	0.029	0.811
25	migraine with aura	0.483	0.030	0.811
26	MIDAS score at baseline	0.547	0.031	0.884
27	emergency department visit	0.621	0.032	0.917
28	average duration of migraine attack	0.622	0.033	0.917
29	MSQ score at baseline	0.633	0.035	0.917
30	pressure	0.679	0.036	0.951
31	chronification	0.728	0.037	0.952
32	throbbing	0.747	0.038	0.952
33	visual changes	0.807	0.039	0.952
34	vise-like	0.817	0.040	0.952
35	dizziness	0.823	0.042	0.952
36	MIDAS-B at follow-up	0.854	0.043	0.952
37	sensory aura	0.868	0.044	0.952
38	MIDAS-B at baseline	0.883	0.045	0.952
39	dull aching	0.903	0.046	0.952
40	severity at follow-up	0.933	0.048	0.952

41	frequency at follow-up	0.939	0.049	0.952
42	average duration of aura	0.952	0.050	0.952

[a] $p < 0.05$ in bold; associations significant at Q = 0.05 in blue

Table S3. Measures/symptoms differing across deciles of PGS004799 in unadjusted analyses

	Decile 1	Deciles 2-9	Decile 10	Total	$\chi^2(2)$, p^a
Photophobia, N (%)					
absent	30 (18.1)	161 (12.2)	15 (9.0)	206 (12.5)	6.67, **0.036**
present	136 (81.9)	1,160 (87.8)	151 (91.0)	1,447 (87.5)	
Stabbing, N(%)					
absent	130 (78.3)	984 (74.5)	107 (64.5)	1,221 (73.9)	9.58, **0.008**
present	36 (21.7)	337 (25.5)	59 (35.5)	432 (26.1)	
Phonophobia, N (%)					
absent	49 (29.5)	322 (24.4)	25 (15.1)	396 (24.0)	10.16, **0.006**
present	117 (70.5)	999 (75.6)	141 (84.9)	1,257 (76.0)	
ln(MIDAS-A), median (range), N	3.0 (0-4.5), 147	3.04 (0-4.5), 1,234	3.26 (1.1-4.5), 154	overall: 7.78, **0.020**<br>decile 1 vs 10: *p*=**0.012**<br>deciles 2-9 vs 10: *p* = **0.023**<br>decile 1 vs 2-9: *p* = 0.349	

[a]χ^2 test for binomial variables, Dunn's test with Sidak correction for continuous variables
